# Robust Radiomics Feature Quantification Using Semiautomatic Volumetric Segmentation

**DOI:** 10.1371/journal.pone.0102107

**Published:** 2014-07-15

**Authors:** Chintan Parmar, Emmanuel Rios Velazquez, Ralph Leijenaar, Mohammed Jermoumi, Sara Carvalho, Raymond H. Mak, Sushmita Mitra, B. Uma Shankar, Ron Kikinis, Benjamin Haibe-Kains, Philippe Lambin, Hugo J. W. L. Aerts

**Affiliations:** 1 Department of Radiation Oncology, Dana-Farber Cancer Institute, Brigham and Women's Hospital, Harvard Medical School, Boston, Massachusetts, United States of America; 2 Department of Radiation Oncology (MAASTRO), Maastricht University, Maastricht, The Netherlands; 3 Machine Intelligence Unit, Indian Statistical Institute, Kolkata, India; 4 University of Massachusetts, Lowell, Massachusetts, United States of America; 5 Department of Radiology, Brigham and Women's Hospital, Harvard Medical School, Boston, Massachusetts, United States of America; 6 Princess Margaret Cancer Centre, University Health Network, Toronto, Ontario, Canada; 7 Department of Medical Biophysics, University of Toronto, Toronto, Ontario, Canada; Northwestern University Feinberg School of Medicine, United States of America

## Abstract

Due to advances in the acquisition and analysis of medical imaging, it is currently possible to quantify the tumor phenotype. The emerging field of Radiomics addresses this issue by converting medical images into minable data by extracting a large number of quantitative imaging features. One of the main challenges of Radiomics is tumor segmentation. Where manual delineation is time consuming and prone to inter-observer variability, it has been shown that semi-automated approaches are fast and reduce inter-observer variability. In this study, a semiautomatic region growing volumetric segmentation algorithm, implemented in the free and publicly available 3D-Slicer platform, was investigated in terms of its robustness for quantitative imaging feature extraction. Fifty-six 3D-radiomic features, quantifying phenotypic differences based on tumor intensity, shape and texture, were extracted from the computed tomography images of twenty lung cancer patients. These radiomic features were derived from the 3D-tumor volumes defined by three independent observers twice using 3D-Slicer, and compared to manual slice-by-slice delineations of five independent physicians in terms of intra-class correlation coefficient (ICC) and feature range. Radiomic features extracted from 3D-Slicer segmentations had significantly higher reproducibility (ICC = 0.85±0.15, p = 0.0009) compared to the features extracted from the manual segmentations (ICC = 0.77±0.17). Furthermore, we found that features extracted from 3D-Slicer segmentations were more robust, as the range was significantly smaller across observers (p = 3.819e-07), and overlapping with the feature ranges extracted from manual contouring (boundary lower: p = 0.007, higher: p = 5.863e-06). Our results show that 3D-Slicer segmented tumor volumes provide a better alternative to the manual delineation for feature quantification, as they yield more reproducible imaging descriptors. Therefore, 3D-Slicer can be employed for quantitative image feature extraction and image data mining research in large patient cohorts.

## Introduction

Lung cancer affects approximately 1.6 million people worldwide every year [1]. The majority of lung cancer cases are non-small cell lung cancer (NSCLC), which has substantially poor prognosis and low survival rates [2].

Medical imaging is one of the major disciplines involved in oncologic science and treatment. By assessing human tissues non-invasively, imaging is extensively used for the detection, diagnosis, staging, and management of lung cancer. Due to the emergence of personalized medicine and targeted treatment, the requirement of quantitative image analysis has risen along with the increasing availability of medical data. Radiomics addresses this issue, and refers to the high throughput extraction of a large number of quantitative and minable imaging features, assuming that these features convey prognostic and predictive information [3], [4]. It focuses on optimizing quantitative imaging feature extraction through computational approaches and developing decision support systems, to accurately estimate patient risk and improve individualized treatment selection and monitoring.

Quantitative imaging features, extracted from medical images, are being extensively examined in clinical research. Several studies have shown the importance of imaging features for treatment monitoring and outcome prediction in lung and other cancer types [5]–[7]. For example, Ganeshan et al. assessed tumor heterogeneity in terms of imaging features extracted from routine computed tomography (CT) imaging in NSCLC, and reported their association with tumor stage, metabolism [8], hypoxia, angiogenesis [9] and patient survival [10]. Furthermore, several studies have uncovered the underlying correlation between gene expression profiles and radiographic imaging phenotype [11], [12]. This kind of radiogenomic analysis has raised the utility of medical image descriptors in clinical oncology by projecting them as potential predictive biomarkers [13], [14].

To ensure the reliability of quantitative imaging features, accurate and robust tumor delineation is essential. Tumor segmentation is one of the main challenges of Radiomics, as manual delineation is prone to high inter-observer variability and represents a time-consuming task [3], [4]. This makes the requirement of (semi)automatic and efficient segmentation methods evident. It has been shown that semiautomatic tumor delineation methods are better alternatives to manual delineations [15], [16]. Recently, we have shown that for NSCLC, semiautomatic segmentation using 3D-Slicer (a free open source software platform for biomedical imaging research) reduces inter-observer variability and delineation uncertainty, compared to manual segmentation [17]. During the evaluation of quantitative imaging features as prognostic or predictive factors, it is essential to determine their variability with respect to the tumor delineation process. We hypothesize that quantitative imaging features extracted from semi-automatically segmented tumors have lower variability and are more robust compared to features extracted from manual tumor delineations, a step forward towards reproducible imaging based models.

In this study we analyzed the robustness of imaging features derived from semi-automatically and manually segmented primary NSCLC tumors in twenty patients. We extracted fifty-six CT 3D-Radiomic features from 3D-Slicer segmentations made by three independent observers, twice, and compared them to the features extracted from manual delineations provided by five independent physicians. As 3D-Slicer is publicly available and easily accessible by download, it can have a large application in Radiomics to extract robust quantitative image features, and be employed for high-throughput data mining research of medical imaging in clinical oncology.

## Results

**Figure 1:**
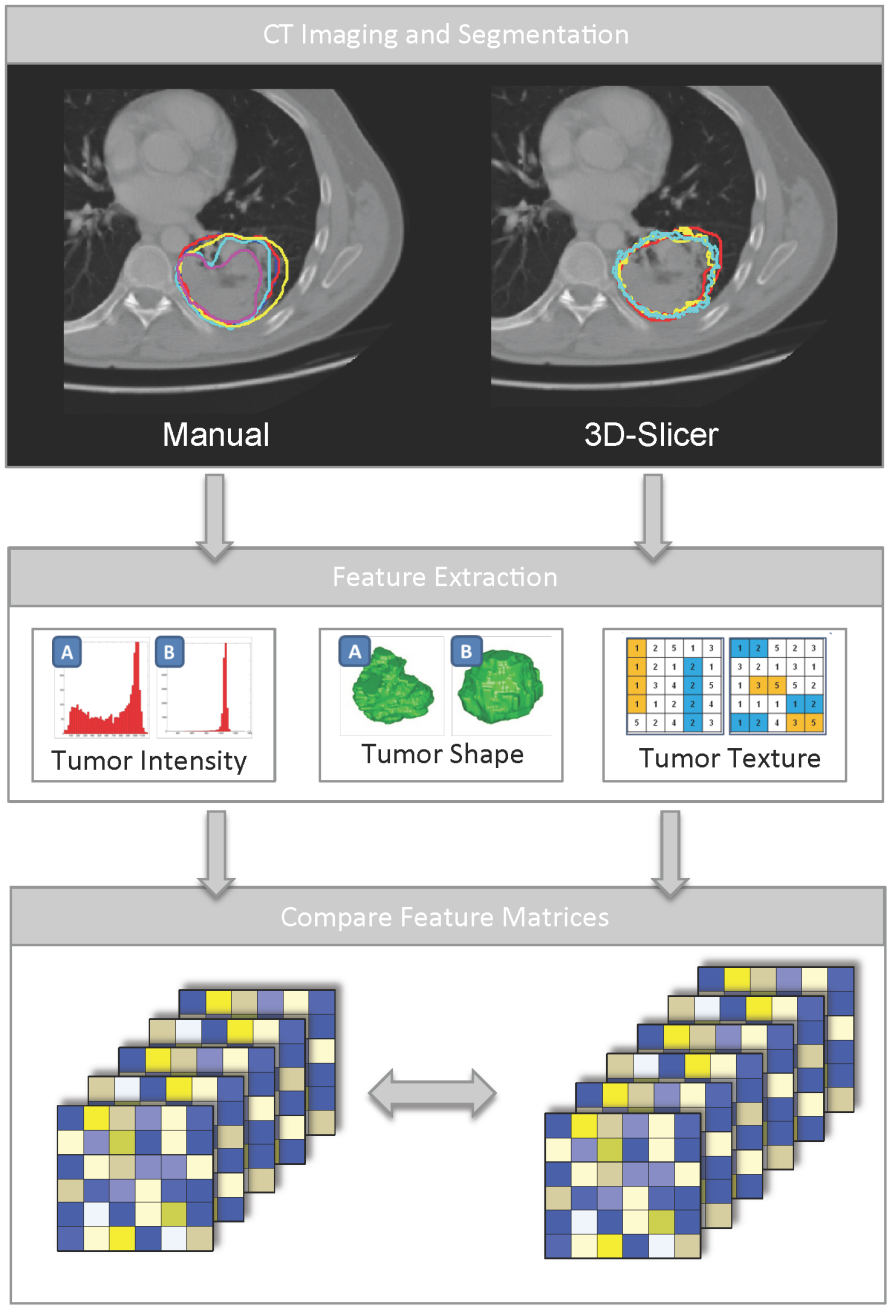
Schematic diagram depicting the overview of the analysis. A: First, we performed five manual delineations and six 3D-Slicer segmentations (three observers twice) on twenty lung tumors. B: Second, fifty-six radiomic features quantifying tumor intensity, texture and shape were extracted from these segmentations. C: Third, the resulting feature matrices were compared for robustness of the feature values.

In order to assess the robustness of 3D-Slicer segmentation on CT imaging for quantitative image feature extraction, we assessed fifty-six 3D-radiomic features quantifying I) tumor intensity, II) tumor shape, and III) tumor texture (**Fig. 1** and Supplement S1). From twenty-lung cancer patients we extracted the radiomic features from 3D-volumes defined by three independent observers twice using 3D-Slicer, and compared them to manual delineations by five independent radiation oncologists.

**Figure 2:**
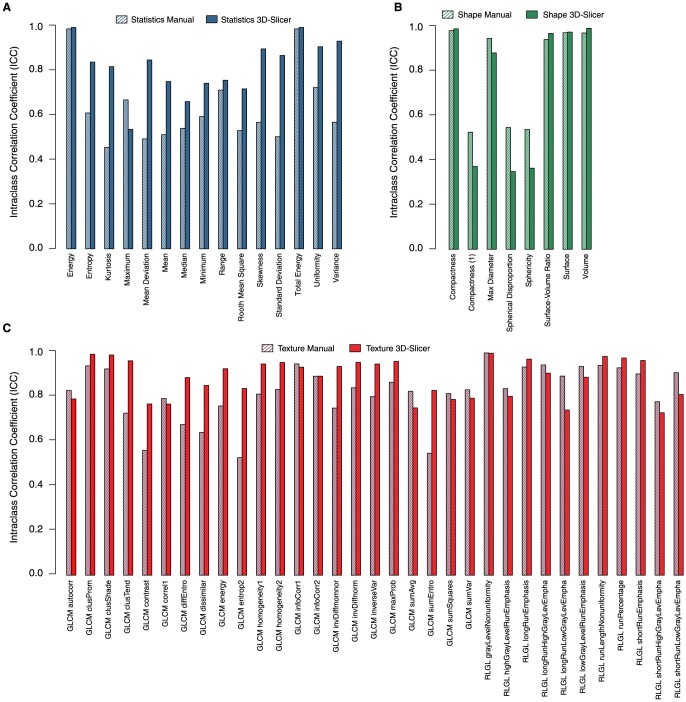
Feature wise comparison of Intra-class correlation coefficients (ICC) between manual and 3D-Slicer segmentations. A: First order statistics features. B: Shape based features. C: Textural features.

**Figure 3:**
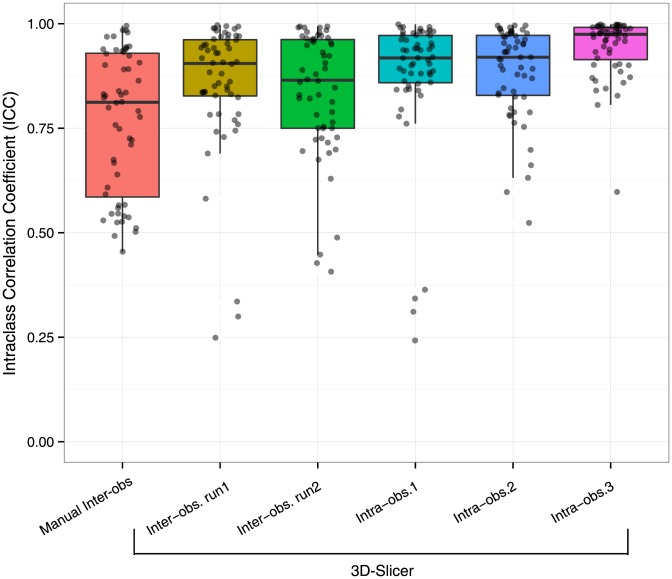
Box-plot comparing intra- and inter-observer reproducibility (ICC) of radiomic features. High inter- and intra- observer reproducibility (ICC) was observed for 3D-Slicer segmentations compared to the inter-observer reproducibility (ICC) of manual delineations. From left the first box refers to the manual inter-observer reproducibility (ICC), second and third boxes refer to the inter-observer reproducibility (ICC) of two different 3D-Slicer segmentation runs. Remaining three boxes refer to the intra-observer reproducibility (ICC) of 3D-Slicer segmentations.

Since two 3D-Slicer segmentations from each of the three observers were considered for the analysis, the six 3D-Slicer segmentations were divided in to two sets, each having three segmentations (one from each observer). We calculated the intra-class correlation coefficient (ICC) for the radiomic features extracted from these two sets of three 3D-Slicer segmentations and five manual delineations. We observed that the radiomic features extracted from 3D-Slicer segmentations, had significantly higher reproducibility (avg. of two 3D-Slicer segmentation sets ICC = 0.85±0.15) as compared to the features extracted from the manual segmentations (ICC = 0.77±0.17) (p = 0.0009, **Fig. 2**). Overall 38 out of the 56 features (68%) showed higher ICC values for 3D-Slicer segmentations as compared to the manual ones. ICC values of all the assessed features are reported in Supplement S2. To evaluate the robustness against multiple algorithmic initializations of the same observer, we computed ICC for the three intra-observer 3D-Slicer segmentation sets, each having two 3D-Slicer segmentations from the same observer. High ICC values (avg. of three intra-observer 3D-Slicer segmentation sets ICC = 0.90±0.17) were observed for intra-observer segmentation groups. **Fig. 3** depicts the ICC values corresponding to the inter-observer manual delineations and intra- & inter-observer 3D-Slicer segmentations.

Intensity statistics and textural features showed significantly higher reproducibility (two sided Wilcoxon test p = 0.0006, p = 0.009, respectively) for 3D-Slicer based segmentations (avg. inter-observer ICC = 0.82±0.13, ICC = 0.88±0.09, respectively) as compared to manual delineations (ICC = 0.63±0.16, ICC = 0.82±0.12, respectively). No statistically significant difference (two sided Wilcoxon test p = 0.31) was observed in ICC values for shape based features between the manual (ICC = 0.80±0.22) and semiautomatic (avg. inter-observer ICC = 0.75±0.31) groups. Fourteen out of 15 statistical features (93%), and 20 out of 33 textural features (67%), showed higher reproducibility (higher ICC) for 3D-Slicer segmentations as compared to manual delineations. For shape based descriptors there was no clear winner between the two segmentation strategies as 4 out of 8 (50%) features turned out having higher ICC for 3D-Slicer segmentations.

We next classified the 56 features into three groups according to their ICC values, as (I) having a high (ICC≥0.8), (II) medium (0.8>ICC≥0.5), or (III) low (ICC<0.5) reproducibility (Supplement S2). For manual delineations, 52% of all the assessed features had high, 45% had medium, and 3% had low reproducibility on the other hand for 3D-Slicer based semiautomatic segmentations, 70% features had high, 25% had medium, and 5% had low reproducibility. Therefore, reproducibility of the features was, in general, higher for 3D-Slicer segmentations.

**Figure 4:**
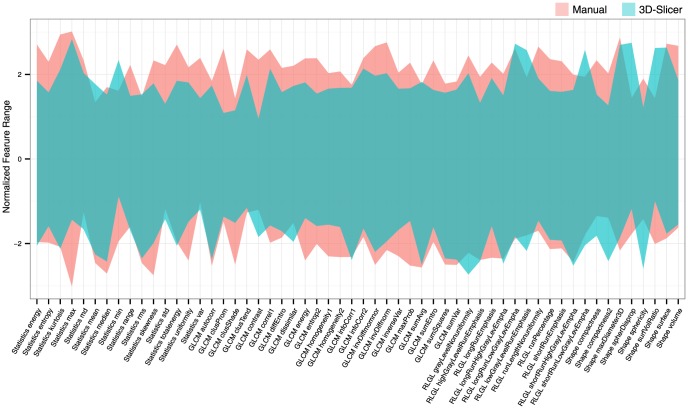
Comparison of normalized feature range between manual and 3D-Slicer segmentation groups. Radiomic features derived from 3D-Slicer segmentations had significantly smaller and overlapping range compared to that from manual delineations.

Furthermore, it becomes important to determine whether the features extracted from semiautomatic segmentations capture the same tumor image properties as with manual delineations. Therefore, we compared the normalized range for all features between these two segmentation groups (**Fig. 4**). We normalized every feature value with respect to all 11 (5 manual+6 3D-Slicer) segmentations, using Z-score normalization. We observed that the features extracted from 3D-Slicer based segmentations, spread over significantly smaller range across observers as compared to those of the manual delineations (two sided Wilcoxon test p = 3.819e-07). Moreover, the features derived from 3D-Slicer segmentations overlapped in range with those of the manual delineations, as the lower(higher) limit(s) being significantly higher(lower) for the 3D-Slicer features (two sided Wilcoxon test p = 0.007, p = 5.863e-06). This corroborates that the feature set, extracted from both the semiautomatic and manual strategies, correspond to similar tumor image characteristics, with the features from 3D-Slicer having less variability across observers.

## Discussion

Medical imaging is considered as one of the fundamental building blocks of clinical oncology. It is routinely used for cancer staging, treatment planning, and treatment response monitoring. Furthermore, recent developments in computational imaging, data mining and predictive analysis have broadened the scope of the imaging in clinical oncology. For example, quantitative imaging features extracted from CT images have been shown to predict 78% of the gene expression variability in hepatocellular carcinoma [11]. In a similar study, image descriptors, extracted from contrast enhanced MRI images of glioblastoma patients, predicted immunohistochemical identified protein expression patterns [12], [18]. Recent computational approaches for image quantification, such as Radiomics, hypothesize that image descriptors extracted from tumor regions are associated with the risk of adverse events after treatment and could provide improved prognostic information for patient management [3], [4].

Accurate and efficient tumor segmentation is one the main challenges for the extraction of robust quantitative imaging features [4]. Manual segmentation suffers from high inter-observer variability and is time consuming [19]. It has been reported that semiautomatic segmentation strategies, as compared to manual delineation can improve tumor segmentation by reducing uncertainty as well as time [15], [17], [19]. These studies focused on tumor volumes while comparing semiautomatic and manual segmentation methods. However, tumor segmentation should also be evaluated in terms of the reliability of radiomic features derived from the volume of interest (VOI), to be used subsequently in prognostic or predictive models.

In this study, we investigated the robustness of quantitative imaging features, extracted from 3D-Slicer tumor segmentations, as compared to those, extracted from manual tumor delineations. Overall 3D-Slicer based semiautomatic segmentation method produced more reproducible radiomic features (p = 0.0009). We also analyzed different feature groups for their reproducibility, and observed that the difference in ICC, for intensity statistics and textural features, was statistically significant (p = 0.0006, p = 0.0094, respectively) between the two segmentation strategies. The shape features, however did not significantly differ in reproducibility between the two strategies (p = 0.31).

We believe the reason for this is that the semiautomatic segmentation covers in more detail the tumor shape, outlining subtle details on shape irregularity i.e. small spiculations in the tumor surface. This may introduce shape irregularities that are not robust between multiple segmentation attempts. Manual contours are usually smoother, by manually contouring a tumor those subtle shape details are smoothed out, so the effect of varying shape is less. Surface area and volume are not be affected by this issue, and that is a reason why they have high ICC values, even higher than manual delineations.

We also analyzed intra– and inter–observer reproducibility for 3D Slicer based semiautomatic segmentations. Three independent observers segmented each tumor twice, with different algorithmic initialization. Image descriptors demonstrated high intra-observer reproducibility for 3D-Slicer segmentations, which indicates their robustness over different seed point initializations. We also observed high inter-observer reproducibility in image descriptors for semiautomatic segmentations. Further reduction of inter-observer variability could be achieved by improving the semiautomatic segmentation strategy, i.e., by reducing observer interaction. Fully automatic methods requiring minimum user interaction, that may solve the complex problem of accurately defining the tumor boundaries, particularly in the case of large tumors with pleural attachment, are still a matter of investigation [20]. Although, current investigation shows that 3D-Slicer segmentation provides a more robust alternative to manual contouring. Furthermore, as 3D-Slicer is publicly available and easily accessible by download, we expect its large utility in the field of quantitative imaging.

Recently the reproducibility of quantitative image features has been evaluated against repetitive test-retest CT image scans, acquired within fifteen minutes time interval, and was used to select the most informative radiomic features [4]. This work was expanded by Hunter et al, to evaluate the robustness of CT image features over three different imaging machines for identifying high quality multi-machine robust radiomic features [21]. In both these studies, since the NSCLC tumors were segmented by a single observer (by using a semiautomatic segmentation), the inter-observer reproducibility of the imaging features could not be evaluated. Leijenaar et al, have analyzed the stability of FDG-PET image features with respect to test-retest scans and inter-observer delineations independently and reported a strong correlation between them [22]. Although they quantified the PET-based radiomic features for manual delineation stability, they did not compare it with that of semiautomatic tumor segmentations. No previous study, in our knowledge, has evaluated the reproducibility of quantitative CT-based imaging features in NSCLC, with respect to tumor segmentation methods.

One of the limitations of our study is not being able to associate these image descriptors with patient outcome due to cohort size and unavailability of clinical data. It would be interesting to investigate the effects of manual and semiautomatic segmentations on the image descriptor based prognostic performance. However, in a recent study, we evaluated the importance of these features for prognosis [23]. A larger number of imaging features showed prognostic performance for both lung and head and neck cancer patients. The scope of the present study was to evaluate feature reproducibility using semiautomatic and manual segmentation techniques. Based on the results presented here, we anticipate that the prognostic performance of imaging markers is likely to increase by using semiautomatic segmentation. For validating the clinical utility further, future studies have to evaluate semiautomatic segmentation vs. manual in terms of prognostic or predictive performance of imaging features in large prospective cohorts.

Besides segmentation methods, other sources of variation should also be considered while evaluating quantitative image features. For instance, Galavis et al. investigated the variability in quantitative image descriptors due to different image acquisition modes and reconstruction parameters [24]. It has also been shown that different ways of image discretization influence the variability of textural features [25]. Although image acquisition, reconstruction and delineation protocols are typically standardized in the clinical practice, there still exists significant variation between imaging studies. Standardized protocols using semiautomatic segmentation tools are also warranted. Therefore, imaging features should be selected based on their robustness towards these sources of variation as well as their prognostic performance.

In conclusion, 3D-Slicer based semiautomatic segmentation significantly improves the robustness of radiomic feature quantification and thus could serve as a potential alternative to the time consuming manual segmentation process. 3D-Slicer can have a large application in radiomic research to extract robust quantitative image features, and be employed for high-throughput data mining research of medical imaging in clinical oncology.

## Methods

### CT-PET scans of NSCLC patients

The imaging data was acquired at MAASTRO Clinic in The Netherlands, as reported previously by Baardwijk et al [26]. In short, twenty patients with histologically verified non-small cell lung cancer, stage IB-IIIB, were included in this study. All patients received a diagnostic whole body positron emission tomography (PET)-computed tomography (CT) scan (Biograph, SOMATOM Sensation 16 with an ECAT ACCEL PET scanner; Siemens, Erlangen, Germany). Patients were instructed to fast at least six hours before administration of ^18^F-fluoro-2-deoxy-glucose (FDG) (MDS Nordion, Liège, Belgium), followed by physiologic saline (10 mL). After the injection of FDG, the patients were encouraged to rest for a period of 45 minutes. Next, free-breathing PET and CT images were acquired. The whole thorax spiral CT scan was acquired with intravenous contrast. The PET images were obtained in 5-min bed positions. The complete data set was then reconstructed iteratively with a reconstruction increment of 5 mm. This study was approved by the local Medical Ethics Committee (Maastricht University Medical Center) and according to the Dutch law. As it was a retrospective study the requirement for informed consent was waived. Imaging data are available on www.cancerdata.org [27].

### Semiautomatic segmentation in 3D slicer

For the semiautomatic segmentation, the GrowCut algorithm implemented in 3D-Slicer was used (www.slicer.org). GrowCut is an interactive region growing segmentation strategy. Given an initial set of label points the algorithm automatically segments the remaining image by using cellular automation. The algorithm uses a competitive region growing approach and is considered to provide good accuracy and speed for both the 2D and 3D image segmentation. For N-class segmentation the algorithm needs N initial sets of labeled pixels (one set corresponding to each class) from the user. Based on these, the algorithm automatically generates the region of interest (ROI), which is the convex hull of the user-labeled pixels with an additional margin. Next, it iteratively labels all the remaining pixels in the ROI using user-given pixel labels. Pixel labeling is done using a weighted similarity score, which is a function of the neighboring pixel weights. An unlabeled pixel is labeled corresponding to the neighboring pixels that have the highest weights. The algorithm converges when all the pixels in the ROI have unchanged labels across several iterations.

3D-Slicer provides a graphical user interface (GUI) as the frontend and an efficient algorithm as the backend for the GrowCut segmentation. After loading the patient data, the process begins with the user initialization of the foreground and background by manually marking the area inside and outside the tumor region. Next, the Growcut automatic competing region-growing algorithm gets activated, and segments the ROI into foreground and background regions. Thereafter, background and the surrounding isolated foreground pixels are removed following visual inspection. If needed, the foreground tumor can be manually edited in a finalization phase. This is a semi-automatic segmentation algorithm because it involves user definition of tumor and background as well as optional manual editing of the final contour.

### Manual Tumor Delineations

Five physicians manually delineated the gross tumor volume (GTV) of the primary tumor based on fused PET-CT images using standard delineation protocol [which includes fixed window-level settings of both CT (lung W 1,700; L −300, mediastinum W 600; L 40) and PET scan (W 30,000; L 15,000) [2], [26]. Radiation oncologists were mutually blind of each other's delineations. The primary GTV was defined for each patient based on combined CT and PET information along the axial plane. The physicians were given transversal, coronal, sagittal and 3D views simultaneously. A treatment planning system (XiO; Computer Medical System, Inc., St. Louis, MO) was used for performing delineations.

### Image processing and feature extraction

All image data were loaded and analyzed in Matlab R2012b (The Mathworks, Natick, MA) using an adapted version of CERR (Computational Environment for Radiotherapy Research) [28], extended with in-house developed Radiomics image analysis software to extract imaging features.

From the five manual and the six 3D-Slicer segmentations, we extracted fifty-six 3D-Radiomic features for the computed tomography scans. See **Fig. 1** for an illustration of the employed methodology. A mathematical description of all features is shown in Supplement S1. The radiomic features were divided in three groups: (I) tumor intensity, (II) shape, and (III) texture. The tumor intensity features consisted of features describing histogram of voxel intensity values contained within the volume of interest (VOI). Shape features were calculated, describing the three-dimensional shape and size of the lesions. Textural features describing patterns or spatial distribution of voxel intensities, were calculated from gray level co-occurrence (GLCM) [29] and gray level run-length (GLRLM) matrices respectively [30]. Determining texture matrix representations requires the voxel intensity values within the VOI to be discretized. This step not only reduces image noise, but also normalizes intensities across all patients, allowing for a direct comparison of all calculated textural features between patients. Texture matrices were determined considering 26-connected voxels (i.e. voxels were considered to be neighbors in all 13 symmetric directions in three dimensions), and a distance of one voxel between consecutive voxels was set for computing co-occurrence and gray level run-length matrices. Features derived from co-occurrence and gray level run-length matrices were calculated by averaging their value over all 13 considered symmetric directions in three dimensions. Overall, the extracted imaging features comprised 15 features describing tumor intensity, 8 shape features and 33 textural features.

### Statistical analysis

Intra-class correlation coefficient (ICC) was calculated in order to quantify the feature reproducibility. The ICC is a statistical measure, ranging between 0 and 1, indicating null and perfect reproducibility, respectively. In order to determine the ICC for inter-observer segmentations, variance estimates were obtained from two-way mixed effect model of analysis of variance (ANOVA). McGraw and Wong [31] defined ICC in case 3A to measure the absolute agreement as,
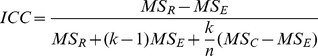
ICC values for intra-observer segmentations were obtained from one-way analysis of variance (ANOVA). It is defined using case 1 of McGraw and Wong [31] as,
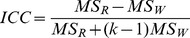
Where 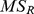 = mean square for rows, 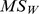 = mean square for residual sources of variance, 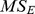 = mean square error, 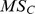 = mean square for columns, 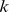 = number of observers involved and 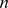 = number of subjects. R package IRR (inter rater reliability) was used for ICC computation [32].

Wilcoxon rank-sum test was used to compare the reproducibility of image features derived from manual and 3D-Slicer segmentations methods. Two methods were considered to be significantly different when the p-value was lower than 0.05. All data are expressed as mean ± SD. All the analyses were performed in Matlab (The MathWorks Inc., Natick, MA, USA) and R (R Foundation for Statistical Computing, Vienna, Austria).

## Supporting Information

Supplement S1 **Mathematical definitions of imaging features.**
(PDF)

Supplement S2 **Results for the reproducibility analysis, showing ICC for radiomic features, derived from manual and 3D-Slicer segmentations, as well as feature reproducibility class, defined as high (ICC≥0.8), medium (0.8>ICC≥0.5), or low (ICC<0.5).**
(PDF)
