# Supplementary material for: Robust Radiomics Feature Quantification Using Semiautomatic Volumetric Segmentation
Source: PLoS One. 2014 Jul 15;9(7):e102107. doi: 10.1371/journal.pone.0102107 (PMC4098900; doi:10.1371/journal.pone.0102107)
Supplement: Supplement S1 — Mathematical definitions of imaging features. (PDF) [file pone.0102107.s001.pdf]

Supplements

Robust Radiomics Feature Quantification using Semiautomatic Volumetric Segmentation

Chintan Parmar, Emmanuel Rios Velazquez, Ralph Leijenaar, Mohammed Jermoumi, Sara Carvalho, Raymond H. Mak, Sushmita Mitra, B. Uma Shankar, Ron Kikinis, Benjamin Haibe-Kains, Philippe Lambin, Hugo J.W.L. Aerts

Supplement SI: Definition of imaging features

First-order intensity statistics based features:

First-order statistics describe the gray level distribution of the image. The following features are calculated: minimum, maximum, range, mean, standard deviation, variance, median, skewness, kurtosis, entropy, root mean square (RMS) and total energy.

- **Energy**

$$E = \sum_{x=1}^{X}\sum_{y=1}^{Y}\sum_{z=1}^{Z} I(x,y,z)^2$$

- **Entropy**

$$H = -\sum_{i=1}^{N_g} P(i)\, log_2\, P(i)$$

Where *P* is the first order histogram and *P*(*i*) the fraction of pixels with gray level *i* .

- **Kurtosis**

$$\gamma_2 = \frac{1}{XYZ}\sum_{x=1}^{X}\sum_{y=1}^{Y}\sum_{z=1}^{Z}\left\{\left[\frac{I(x,y,z)-\mu}{\sigma}\right]\right\}^4 - 3$$

The sharpness of the histogram is described by the kurtosis

- **Maximum**

$$I_{max} = \max\{I(x,y,z)\}$$

It describes the maximum CT value.

- **Mean Deviation**

$$md = \frac{1}{XYZ}\sum_{x=1}^{X}\sum_{y=1}^{Y}\sum_{z=1}^{Z}|(I(x,y,z)-\mu)|$$

It is called as mean absolute deviation.

- **Mean**

$$\mu = \frac{1}{XYZ}\sum_{x=1}^{X}\sum_{y=1}^{Y}\sum_{z=1}^{Z} I(x,y,z)$$

Average CT value.

- **Median**

The median is the value that separates the lower and upper half of the sorted array of pixel values.

- **Minimum**

$$I_{min} = \min\{I(x,y,z)\}$$

It describes the maximum CT value

- **Range**

$$R = \max\{I(x,y,z)\} - \min\{I(x,y,z)\}$$

It describes the range of the CT value.

- **Root mean square (RMS)**

$$RMS = \sqrt{\frac{1}{XYZ}\sum_{x=1}^{X}\sum_{y=1}^{Y}\sum_{z=1}^{Z} I(x,y,z)^2}$$

The RMS is the square root of the average of the sum of all pixel values squared. In contrast to the mean, the RMS value is not affected by a pixel value being positive or negative.

- **Skewness**

$$\gamma_1 = \frac{1}{XYZ}\sum_{x=1}^{X}\sum_{y=1}^{Y}\sum_{z=1}^{Z}\left[\frac{I(x,y,z)-\mu}{\sigma}\right]^3$$

The degree of asymmetry around the mean value is described by the skewness

Ng is the number of discrete gray levels. Entropy is a measure of (non)-uniformity of the histogram. Very low entropy would indicate that the pixels of the image all have roughly the same value. Higher entropy means the histogram is closer to a uniform distribution.

- **Standard deviation**

$$S = \sqrt{\frac{1}{(XYZ-1)}\sum_{x=1}^{X}\sum_{y=1}^{Y}\sum_{z=1}^{Z}(I(x,y,z)-\mu)^2}$$

- **Total energy (multiplied by the voxel volume)**

$$E_{tot} = V_{voxel}\sum_{x=1}^{X}\sum_{y=1}^{Y}\sum_{z=1}^{Z} I(x,y,z)^2$$

Where V_{voxel} is the voxel volume of the three dimensional image. The voxel volume is the product of the pixel spacing in x-direction, the pixel spacing in y-direction and the pixel spacing in z-direction.

- **Uniformity**

$$U = \sum_{i=1}^{N_g} P(i)^2$$

Where P is the first order histogram and $P(i)$ the fraction of pixels with gray level i .

N_g is the number of discrete gray levels. It describes the uniformity of the Image.

- **Variance**

$$\sigma^2 = \frac{1}{(XYZ-1)} \sum_{x=1}^{X} \sum_{y=1}^{Y} \sum_{z=1}^{Z} (I(x,y,z) - \mu)^2$$

Shape based features:

These features provide more insight in the shape and size of the tumor volume of interest. Shape based features are: volume, surface area, surface to volume ratio, sphericity, spherical disproportion, compactness and maximum 3D diameter.

- **Compactness**

$$Comp = \frac{V}{\sqrt{\pi}\, A^{\frac{2}{3}}}$$

- **Compactness (1)**

$$Comp1 = 36\pi \, \frac{V^2}{A^3}$$

Compactness, as the name already states, indicates how compact a 3D shape is. The most compact shape is a perfect sphere.

- **Maximum diameter 3D**

The maximum diameter is the Euclidean distance between the two voxels that lie farthest apart.

With each voxel *i* having coordinate $(x_i, y_{i,} z_i)$, the Euclidean distance between two voxels is described as:

$$Dist = \sqrt{(x_2 - x_1)^2 + (y_2 - y_1)^2 + (z_2 - z_1)^2}$$

- **Spherical disproportion**

$$\varphi_D = \frac{A}{4\pi R^2}$$

Where,

$$R = \left(\frac{3V}{4\pi}\right)^{\frac{1}{3}}$$

R is the radius of the sphere with the same volume as the tumor. Spherical disproportion indicates how close the shape is to a sphere.

- **Sphericity**

Sphericity describes how spherical, or rounded, the shape of the tumor is.

It is defined as the surface area of a sphere, having the same volume as the tumor, divided by the surface area of the tumor.

$$\varphi = \frac{(6V)^{\frac{2}{3}}\pi^{\frac{1}{3}}}{A}$$

- **Surface area**

To calculate the surface area, the area is divided into 3D triangles. The surface area then is:

$$A = \sum_{i=1}^{N_{t\,3D}} B_i$$

Where $N_{t\,3D}$ is the total number of 3D triangles and $B_i = \frac{1}{2}\left|V_{i,1}V_{i,2} * V_{i,1}V_{i,3}\right|$

Where B_i is the surface area of one 3D triangle and $V_{i,(1,2,3)}$ an edge vector of the 3D triangle.

- **Volume**

$$V = V_{voxel} * N_{voxels}$$

The volume is calculated as the total number of voxels N_{voxels} multiplied by the voxel volume V_{voxel}.

The voxel volume is:

$$V_{voxel} = \Delta x * \Delta y * \Delta z$$

Where Δx, Δy, Δz are pixel spacing in x, y and z direction

- **Surface to volume ratio**

The surface to volume ratio is a measure to quantify how lobulated the tumor volume is.

$$SV = \frac{A}{V}$$

Tumor with higher lobulated volume has a higher surface to volume ratio.

Texture features:

Gray-Level Co-Occurrence Matrix based features

Gray level co-occurrence matrix based features, as described by Haralick et al. Let:

$P(i, j)$ be the co-occurrence matrix,

N_g be the number of discrete intensity levels in the image,

μ be the mean of $P(i, j)$,

$\mu_x(i)$ be the mean of row i,

$\mu_y(j)$ be the mean of column j,

$\sigma_x(i)$ be the standard deviation of row i,

$\sigma_y(j)$ be the standard deviation of column j,

$p_x(i) = \sum_{j=1}^{N_g} P(i, j)$,

$p_y(i) = \sum_{i=1}^{N_g} P(i, j)$,

$p_{x+y}(k) = \sum_{i=1}^{N_g}\sum_{j=1}^{N_g} P(i,j)$, $i+j=k$, $k = 2,3,\ldots,2N_g$,

$p_{x-y}(k) = \sum_{i=1}^{N_g}\sum_{j=1}^{N_g} P(i,j)$, $|i-j| = k$, $k = 0,1,\ldots,N_g - 1$,

$HXY1 = -\sum_{i=1}^{N_g}\sum_{j=1}^{N_g} P(i,j)\log(p_x(i)p_y(j))$,

$HXY2 = -\sum_{i=1}^{N_g}\sum_{j=1}^{N_g} p_x(i)p_y(j)\log(p_x(i)p_y(j))$.

- **Autocorrelation:**

$$autocorrelation = \sum_{i=1}^{N_g}\sum_{j=1}^{N_g} ijP(i,j)$$

It measures the coarseness of an image and evaluates the linear spatial relationships between texture primitives.

- **Cluster Prominence:**

$$cluster\ prominence = \sum_{i=1}^{N_g}\sum_{j=1}^{N_g}\left[i + j - \mu_x(i) - \mu_y(j)\right]^4 P(i,j)$$

It measures local intensity variation.

- **Cluster Shade:**

$$cluster\ shade = \sum_{i=1}^{N_g}\sum_{j=1}^{N_g}\left[i + j - \mu_x(i) - \mu_y(j)\right]^3 P(i,j)$$

- **Cluster Tendency:**

$$cluster\ tendency = \sum_{i=1}^{N_g}\sum_{j=1}^{N_g}\left[i + j - \mu_x(i) - \mu_y(j)\right]^2 P(i,j)$$

Cluster shade and cluster prominence are measures of the skewness of the matrix, in other words the lack of symmetry. When cluster shade and cluster prominence are high, the image is not symmetric.

- **Contrast:**

$$contrast = \sum_{i=1}^{N_g}\sum_{j=1}^{N_g}|i-j|^2 P(i,j)$$

It measures local intensity variation.

- **Correlation:**

$$correlation = \frac{\sum_{i=1}^{N_g}\sum_{j=1}^{N_g} ijP(i,j) - \mu_i(i)\mu_j(j)}{\sigma_x(i)\sigma_y(j)}$$

Correlation is a measure of gray level linear dependence between the pixels at the specified positions relative to each other.

- **Difference entropy:**

$$difference\ entropy = \sum_{i=0}^{N_g-1} P_{x-y}(i)\log_2\left[P_{x-y}(i)\right]$$

- **Dissimilarity:**

$$dissimilarity = \sum_{i=1}^{N_g}\sum_{j=1}^{N_g}|i-j|P(i,j)$$

- **Energy:**

$$energy = \sum_{i=1}^{N_g}\sum_{j=1}^{N_g}[P(i,j)]^2$$

The energy is a measure of homogeneity of the image. If the image is homogeneous, the image contains only a few gray levels. This results in a GLCM with only a few, but relatively high values of *p(i, j)*. The sum of squares will then be high.

- **Entropy:**

$$entropy = -\sum_{i=1}^{N_g}\sum_{j=1}^{N_g} P(i,j)\log_2[P(i,j)]$$

Inhomogeneous Images have low entropy, while a homogeneous scene has high entropy.

- **Homogeneity 1:**

$$homogeneity\ 1 = \sum_{i=1}^{N_g}\sum_{j=1}^{N_g}\frac{P(i,j)}{1+|i-j|}$$

- **Homogeneity 2:**

$$omogeneity\ 2 = \sum_{i=1}^{N_g}\sum_{j=1}^{N_g}\frac{P(i,j)}{1+|i-j|^2}$$

- **Informational measure of correlation 1 (IMC1):**

$$IMC1 = \frac{H - HXY1}{\max\{HX, HY\}}$$

Where H is the entropy.

- **Informational measure of correlation 2 (IMC2):**

$$IMC2 = \sqrt{1 - e^{-2(HXY2-H)}}$$

Where H is the entropy.

- **Inverse Difference Moment Normalized (IDMN):**

$$IDMN = \sum_{i=1}^{N_g}\sum_{j=1}^{N_g}\frac{P(i,j)}{1+\left(\frac{|i-j|^2}{N^2}\right)}$$

- **Inverse Difference Normalized (IDN):**

$$IDN = \sum_{i=1}^{N_g}\sum_{j=1}^{N_g}\frac{P(i,j)}{1+\left(\frac{|i-j|}{N}\right)}$$

IDMN and IDN measures image homogeneity as it assumes larger values for smaller gray tone differences in pair elements. It is more sensitive to the presence of near diagonal elements in the GLCM. It has maximum value when all elements in the image are same.

- **Inverse variance:**

$$inverse\ variance = \sum_{i=1}^{N_g}\sum_{j=1}^{N_g}\frac{P(i,j)}{|i-j|^2} \quad , i \neq j$$

- **Maximum Probability:**

$$maximum\ probability = \max\{P(i,j)\}$$

- **Sum average:**

$$sum\ average = \sum_{i=2}^{2N_g}[iP_{x+y}(i)]$$

- **Sum entropy:**

$$sum\ entropy = -\sum_{i=2}^{2N_g} P_{x+y}(i) \log_2\left[P_{x+y}(i)\right]$$

- **Sum variance:**

$$sum\ variance = \sum_{i=2}^{2N_g} (i - SE)^2 P_{x+y}(i)$$

- **Variance:**

$$variance = \sum_{i=1}^{N_g}\sum_{j=1}^{N_g} (i-\mu)^2 P(i,j)$$

This feature puts relatively high weights on the elements that differ from the average value of *p(i,j)*.

Gray-Level Run-Length matrix based features

Gray-level run-length matrix based features, as described by Galloway et al. Let:

$p(i,j|\theta)$ be the (i,j)th entry in the given run-length matrix p for a direction θ,

N_g the number of discrete intensity values in the image,

N_r the number of different run lengths,

N_p the number of voxels in the image.

- **Gray Leven Non-Uniformity (GLN)**

$$GLN = \frac{\sum_{i=1}^{N_g}\left[\sum_{j=1}^{N_r} p(i,j|\theta)\right]^2}{\sum_{i=1}^{N_g}\sum_{j=1}^{N_r} p(i,j|\theta)}$$

It measures the similarity of gray level intensity values in the image. The GLN is low if the intensity values are alike.

- **High Gray Level Run Emphasis (HGLRE)**

$$HGLRE = \frac{\sum_{i=1}^{N_g}\sum_{j=1}^{N_r} i^2 p(i,j|\theta)}{\sum_{i=1}^{N_g}\sum_{j=1}^{N_r} p(i,j|\theta)}$$

It measures the distribution of high gray level values. The HGRE is high for the image with high gray level values.

- **Long Run Emphasis (LRE)**

$$LRE = \frac{\sum_{i=1}^{N_g}\sum_{j=1}^{N_r} j^2 p(i,j|\theta)}{\sum_{i=1}^{N_g}\sum_{j=1}^{N_r} p(i,j|\theta)}$$

It measures distribution of long runs. The LRE is highly dependent on the occurrence of long runs and it gives high value for coarse structural textures.

- **Long Run High Gray Level Emphasis (LRHGLE)**

$$LRHGLE = \frac{\sum_{i=1}^{N_g}\sum_{j=1}^{N_r} p(i,j|\theta) i^2 j^2}{\sum_{i=1}^{N_g}\sum_{j=1}^{N_r} p(i,j|\theta)}$$

It measures the joint distribution of long runs and high gray level values. The LRHGE is high for images with many long runs and high gray level values.

- **Long Run Low Gray Level Emphasis (LRLGLE)**

$$LRLGLE = \frac{\sum_{i=1}^{N_g}\sum_{j=1}^{N_r} \left[\frac{p(i,j|\theta) j^2}{i^2}\right]}{\sum_{i=1}^{N_g}\sum_{j=1}^{N_r} p(i,j|\theta)}$$

It measures the joint distribution of long runs and low gray level values. The LRLGE is high for the image with many long runs and low gray level values.

- **Low Gray Level Run Emphasis (LGLRE)**

$$LGLRE = \frac{\sum_{i=1}^{N_g}\sum_{j=1}^{N_r} \left[\frac{p(i,j|\theta)}{i^2}\right]}{\sum_{i=1}^{N_g}\sum_{j=1}^{N_r} p(i,j|\theta)}$$

It measures the distribution of low gray level values. The LGRE is high for the image with low gray level values.

- **Run Length Non-Uniformity (RLN)**

$$RLN = \frac{\sum_{j=1}^{N_r}\left[\sum_{i=1}^{N_g} p(i,j|\theta)\right]^2}{\sum_{i=1}^{N_g}\sum_{j=1}^{N_r} p(i,j|\theta)}$$

It measures the similarity of the length of runs throughout the image. The RLN is low if the run lengths are alike.

- **Run Percentage (RP)**

$$RP = \sum_{i=1}^{N_g}\sum_{j=1}^{N_r}\frac{p(i,j|\theta)}{N_p}$$

It measures the homogeneity and the distribution of runs of an image in the direction. The RP is very high if the all gray levels have the runs of length 1.

- **Short Run Emphasis (SRE)**

$$SRE = \frac{\sum_{i=1}^{N_g}\sum_{j=1}^{N_r}\left[\frac{p(i,j|\theta)}{j^2}\right]}{\sum_{i=1}^{N_g}\sum_{j=1}^{N_r} p(i,j|\theta)}$$

It measures the distribution of short runs. The SRE is highly dependent on the occurrence of short runs and it gives high value for fine texture the value of SRE is high.

- **Short Run High Gray Level Emphasis (SRHGLE)**

$$SRHGLE = \frac{\sum_{i=1}^{N_g}\sum_{j=1}^{N_r}\left[\frac{p(i,j|\theta)i^2}{j^2}\right]}{\sum_{i=1}^{N_g}\sum_{j=1}^{N_r} p(i,j|\theta)}$$

It measures the joint distribution of short runs and high gray level values. The SRHGE is high for the image with many short runs and high gray level values.

- **Short Run Low Gray Level Emphasis (SRLGLE)**

$$SRLGLE = \frac{\sum_{i=1}^{N_g} \sum_{j=1}^{N_r} \left[\frac{p(i,j|\theta)}{i^2 j^2}\right]}{\sum_{i=1}^{N_g} \sum_{j=1}^{N_r} p(i,j|\theta)}$$

It measures the joint distribution of short runs and low gray level values. The SRLGE is high for the image with many short runs and lower gray level values.
