# Supplementary material for: Robust Radiomics Feature Quantification Using Semiautomatic Volumetric Segmentation
Source: PLoS One. 2014 Jul 15;9(7):e102107. doi: 10.1371/journal.pone.0102107 (PMC4098900; doi:10.1371/journal.pone.0102107)
Supplement: Supplement S2 — Results for the reproducibility analysis, showing ICC for radiomic features, derived from manual and 3D-Slicer segmentations, as well as feature reproducibility class, defined as high (ICC≥0.8), medium (0.8>ICC≥0.5), or low (ICC<0.5). (PDF) [file pone.0102107.s002.pdf]

Supplements

Robust Radiomics Feature Quantification using Semiautomatic Volumetric Segmentation

Chintan Parmar, Emmanuel Rios Velazquez, Ralph Leijenaar, Mohammed Jermoumi, Sara Carvalho, Raymond H. Mak, Sushmita Mitra, B. Uma Shankar, Ron Kikinis, Benjamin Haibe-Kains, Philippe Lambin, Hugo J.W.L. Aerts

Supplement SII: Table

Results for the reproducibility analysis, showing ICC for radiomic features, derived from manual and 3D-Slicer segmentations, as well as feature reproducibility class, defined as high (ICC≥0.8), medium (0.8>ICC≥0.5), or low (ICC<0.5).

Feature Name	Manual ICC	Manual Feature Reproducibility Class	3D-Slicer ICC (avg of two inter-obs. sets)	3D Slicer Feature Reproducibility Class
**Intensity statistics based Features (First order)**				
Energy	0.98	High	0.99	High
Entropy	0.61	Medium	0.84	High
Kurtosis	0.45	Low	0.82	High
Maximum	0.67	Medium	0.53	Medium
Mean deviation	0.49	Low	0.85	High
Mean	0.51	Medium	0.75	Medium
Median	0.54	Medium	0.66	Medium
Minimum	0.59	Medium	0.74	Medium
Range	0.71	Medium	0.76	Medium
Root mean square	0.52	Medium	0.72	Medium
Skewness	0.57	Medium	0.90	High
Standard deviation	0.50	Medium	0.87	High
Total Energy	0.98	High	0.99	High
Uniformity	0.72	Medium	0.90	High
Variance	0.57	Medium	0.93	High
**Group (mean ± SD)**	**0.63 ± 0.16**		**0.82 ± 0.13**	
**Shape based Features**				
Compactness 1	0.98	High	0.99	High
Compactness 2	0.52	Medium	0.37	Low
Maximum diameter	0.94	High	0.88	High
Sph. Disproportion	0.64	Medium	0.35	Low
Sphericity	0.54	Medium	0.36	Low
Surface area	0.94	High	0.97	High
Surface/volume	0.97	High	0.97	High
Volume	0.97	High	0.99	High
**Group (mean ± SD)**	**0.80 ± 0.22**		**0.75 ± 0.31**	
**Texture Features (GLCM-GLRL)**				
Autocorrelation	0.83	High	0.79	Medium
Cluster prominence	0.97	High	0.99	High
Cluster shade	0.92	High	0.99	High
Cluster tendency	0.73	Medium	0.96	High
Contrast	0.56	Medium	0.77	High
Correlation	0.79	Medium	0.77	Medium
Difference entropy	0.67	Medium	0.88	High
Dissimilarity	0.64	Medium	0.85	High
Energy	0.76	Medium	0.92	High
Entropy	0.53	Medium	0.84	High
Homogeneity 1	0.81	High	0.95	High
Homogeneity 2	0.83	High	0.95	High
IMC1	0.95	High	0.93	High
IMC2	0.89	High	0.89	High

IDMN	0.75	Medium	0.93	High
IDN	0.84	High	0.95	High
Inverse variance	0.79	Medium	0.95	High
Max. prob.	0.86	High	0.96	High
Sum average	0.82	High	0.75	Medium
Sum entropy	0.55	Medium	0.83	High
Sum variance	0.81	High	0.79	Medium
Variance	0.83	High	0.79	High
GLN	0.99	High	0.99	High
HGLRE	0.84	High	0.80	High
LGLRE	0.93	High	0.97	High
LRE	0.94	High	0.90	High
LRHGLE	0.89	High	0.74	Medium
LRLGLE	0.93	High	0.89	High
RLN	0.94	High	0.98	High
RP	0.93	High	0.97	High
SRE	0.90	High	0.96	High
SRHGLE	0.78	Medium	0.73	Medium
SRLGLE	0.91	High	0.81	High
**Group (mean ± SD)**	**0.82 ± 0.12**		**0.88 ± 0.09**	
**Total (mean ± SD)**	**0.77 ± 0.17**		**0.85 ± 0.15**	

Intensity statistics and textural features were significantly higher (two sided Wilcoxon test p= 0.0006, p= 0.009, respectively) in reproducibility (ICC) for 3D-Slicer segmentations as compared to manual delineations. However, no statistically significant difference in reproducibility could be observed (two sided Wilcoxon test p = 0.31) for shape based features between the two segmentation strategies.
